# Aztreonam-avibactam: an option against carbapenem-resistant Enterobacterales with emerging resistance

**DOI:** 10.1093/pcmedi/pbac029

**Published:** 2022-11-22

**Authors:** Shikai Wu, Zhiyong Zong

**Affiliations:** Center of Infectious Diseases, West China Hospital, Sichuan University, Chengdu 610041, China; Division of Infectious Diseases, State Key Laboratory of Biotherapy, Sichuan University, Chengdu 610041, China; Center of Infectious Diseases, West China Hospital, Sichuan University, Chengdu 610041, China; Division of Infectious Diseases, State Key Laboratory of Biotherapy, Sichuan University, Chengdu 610041, China; Center for Pathogen Research, West China Hospital, Sichuan University, Chengdu 610041, China

Dear Editor,

The Enterobacterales are an order of Gram-negative bacteria comprising a few major human pathogens such as *Escherichia coli* and *Klebsiella pneumoniae*. However, carbapenem-resistant Enterobacterales (CRE) has risen as an urgent threat for human health, leading to high mortality with very limited antimicrobial options. The main mechanism mediating resistance to β-lactams including carbapenems in the Enterobacterales is production of β-lactamases, which are two categories of enzymes capable of hydrolyzing β-lactams: serine β-lactamases and metallo-β-lactamases (MBLs). Avibactam (AVI) is a non-β-lactam β-lactamase inhibitor able to inhibit almost all serine β-lactamases but not MBLs. AVI in combination with ceftazidime (CAZ) has been approved for treating infections caused by CRE but CAZ-AVI has no activities against those producing MBLs. Currently, no MBL inhibitors have been approved for clinical use. Aztreonam (ATM), a monobactam, is stable to the hydrolysis of MBLs, and AVI can protect ATM from the inactivation by serine β-lactamases. The ATM-AVI combination may therefore be a viable choice against CRE producing serine β-lactamases, MBLs and/or both, which have been supported by large-scale *in vitro* susceptibility studies.^1^

At present, ATM-AVI has not been approved for clinical use. Nevertheless, the combination of ATM and CAZ-AVI, both of which are available in clinical settings, can form *de facto* the ATM-AVI combination. An precision medicine approach can be detection of the type of carbapenem-hydrolyzing β-lactamases (serine β-lactamases and/or MBLs) followed by the selection of CAZ-AVI against CRE without producing MBLs or CAZ-AVI in combination with ATM for those producing MBLs. Such ATM-CAZ-AVI combination has been recommended by European Society of Clinical Microbiology and Infectious Diseases (ESCMID) and Infectious Diseases Society of America for treating severe infections caused by MBL-producing CRE.^2^,^3^ Currently available limited data suggest that ATM-CAZ-AVI is commonly associated with lower mortality rate, fewer clinical failure, and shorter length of stay compared with other active agents such as colistin-based combinations.^4–6^ A systematic review^4^ including 91 patients with infections of MBL-producing CRE from 16 case reports, case series, and cohorts has indicated that 79% of them had clinical resolution within 30 days after receiving ATM-CAZ-AVI. A later retrospective study^5^ including 22 patients receiving ATM-CAZ-AVI against MBL-producing CRE showed a 18.2% 30-day mortality.

However, resistance to ATM-AVI in Enterobacterales has also emerged. Notably, both the Clinical and Laboratory Standards Institute (CLSI) and the European Committee on Antimicrobial Susceptibility Testing (EUCAST, https://www.eucast.org) have not specified the breakpoints for defining resistance to ATM-AVI. Nevertheless, the CLSI resistance breakpoint for ATM (minimum inhibitor concentration [MIC] ≥16 mg/L) is commonly applied for ATM-AVI with AVI fixed at 4 mg/L in literature. Studies of mechanisms for resistance to ATM-AVI have been mainly targeted *E. coli* and *K. pneumoniae*. In *E. coli*, penicillin binding protein 3 (PBP3) is the major target of ATM and a four-amino-acid insertion (YRIK, YRIN, YRIP, or TIPY) in PBP3 could render a reduced susceptibility background against ATM.^7^ In such a background, a few β-lactamases able to efficiently hydrolyze ATM, such as AmpC cephalosporinase CMY-42,^7^,^8^ extended-spectrum β-lactamases CTX-M-15 and CTX-M-199, and carbapenemase KPC-21, could evade the protection from AVI and mediate resistance to ATM-AVI (Table 1). In particular, increased production of these β-lactamases in the presence of PBP3 with a four-amino-acid insertion could lead to elevated levels of resistance to ATM-AVI.^7^

**Table 1.** Aztreonam-avibactam-resistant mechanisms in *Escherichia coli* and *Klebsiella pneumonia*.

**Strain**	**Country of isolation**	**Species^a^**	**ST^b^**	**MIC^c^, ATM-AVI, mg/L**	**Resistance mechanism^d^**
035123	China	EC	410	16	PBP3 ins (YRIK) + CMY-42
035148	China	EC	410	128	PBP3 ins (YRIK) + CMY42 overexpression
035166R	China	EC	410	512	PBP3 ins (YRIN) + KPC-21
Survcare058	Germany	EC	405	16	PBP3 ins (YRIK) + CMY-42
Survcare453	Germany	EC	410	16	PBP3 ins (YRIK) + CMY-42
Survcare253	Germany	EC	2851	16	PBP3 ins (YRIN) + CMY-42
ARC3799	India	EC	410	16	PBP3 ins (YRIN) + CMY-42
R-3058	Angola	EC	-	16	PBP3 ins (YRIN) + CMY-42
R-461	France	EC	-	16	PBP3 ins (YRIN) + CMY-42
1114251	Turkey	EC	410	16	PBP3 ins (YRIK) + CMY-42
1126350	Vietnam	EC	617	16	PBP3 ins (YRIN) + CMY-42
WCHEC96200	China	EC	405	16	PBP3 ins (YRIK) + CTX-M-15
PF_19–40f	UK	EC	-	16	BaeS (Q163L), likely with OmpC mutation^e^
PF_19–51e	UK	EC	-	16	BaeS (Y42H), likely with OmpC mutation^e^
PF_19–51n	UK	EC		32	BaeS (G241D), likely with OmpC mutation^e^
PF_19–52d	UK	EC	-	16	BaeR (G23E), likely with OmpC mutation^e^
PF_19–52j	UK	EC		16	BaeS (F159L), likely with OmpC mutation^e^
PF_19–26a	UK	EC	-	16	CMY-44 (Y150C)
91471	China	KP	11	128	KPC-2 overexpression + OmpK alteration^f^
96202	China	KP	11	128	KPC-2 overexpression + OmpK alteration^f^
98180	China	KP	11	64	KPC-2 overexpression + OmpK alteration^f^
98690	China	KP	11	32	KPC-2 overexpression + OmpK alteration^f^
108728	China	KP	11	>128	KPC-2 overexpression + OmpK alteration^f^
108738	China	KP	11	>128	KPC-2 overexpression + OmpK alteration^f^
108783	China	KP	11	32	KPC-2 overexpression + OmpK alteration^f^
109096	China	KP	11	>128	KPC-2 overexpression + OmpK alteration^f^
116216	China	KP	11	64	KPC-2 overexpression + OmpK alteration^f^
1116221	Thailand	KP	273	>16	*acrA* overexpression + DHA-1 + OmpK36 (Y43X)
Kp202_32A	China	KP	-	>256	CMY-16 (Y150S) + ∆OmpK35 + OmpK36 (ins)
Kp214-R150	China	KP	147	128	CMY-16 (Y150S) + OmpK35 (IS*Ecp1*) + OmpK36 (ins)
Kp231-R150	China	KP	377	128	CMY-16 (Y150S) + OmpK35 (IS*1*)
Kp518-R150	China	KP	258	32	CMY-16 (Y150S) + ∆OmpK35
Kp202_64A	China	KP	-	>256	CMY-16 (N346H) + ∆OmpK35 + OmpK36 (ins)
Kp214-R346	China	KP	147	64	CMY-16 (N346H) + OmpK35 (IS*Ecp1*) + OmpK36 (ins)
Kp231-R346	China	KP	377	32	CMY-16 (N346H) + OmpK35 (IS*1*)
Kp518-R346	China	KP	258	16	CMY-16 (N346H) + ∆OmpK35
KP21(*ramR*) *ompK36* pKPC-3 V239G	UK	KP	-	16	*ramR* (frameshift) + ∆OmpK36 + KPC-3 (V239G)

^a^EC,*E. coli*; KP,*K. pneumoniae*.

^b^-, not determined.

^c^MIC, in the presence of 4 mg/L AVI.

^d^ins, insertion of amino acids. IS*1* and IS*Ecp1* are two insertion sequences interrupting OmpK35. ∆, truncation. The letters in parentheses represent amino acid insertion (YRIK or YRIN) or amino acid substitution with the location being indicated (e.g. S106P refers the substitution of S at location 106 by P). BaeS/BaeR, transcription activator of multidrug efflux pump-encoding genes mdtABCD and acrD; CMY, AmpC cephalosporinase; CTX-M, an extended-spectrum β-lactamase; DHA-1, AmpC cephalosporinase; KPC, Klebsiella pneumoniae carbapenemase; OmpK, outer membrane porin; PBP3, penicillin binding protein 3;*ramR*, repressor of ramA related to efflux pump and porins.

^e^The details of OmpK36 mutations are not available for these strains.

^f^OmpK alteration refers to decreased expression of OmpK35 + truncated OmpK37 + absence of OmpK36.

Mechanisms without the involvement of PBP3 have also been identified. Unlike *E. coli*, alteration of penicillin-binding proteins has not been identified in ATM-AVI-resistant *K. pneumoniae*. In contrast, alteration and/or decreased production of outer membrane porins OmpK35 (OmpF), OmpK36 (OmpC), and/or OmpK37 could also provide the reduced susceptibility background against ATM in Enterobacterales, particularly in *K. pneumonia*.^8–10^ Alteration of these porins may result from various mechanisms such as mutation, truncation, insertion/deletion of amino acids, or interruption by insertion sequences,^8–10^ while their decreased production is largely due to mutations in regulator genes such as BaeS-BaeR two-component system and *ramR*, both of which also regulate efflux pumps. In the background of decreased production and/or alteration of porins, overexpression of carbapenemase gene *bla*_KPC-2_ could confer resistance to ATM-AVI and the resistance level correlates to the expression of *bla*_KPC-2_ in *K. pneumonia*.^9^ KPC-3 with a V239G (Ambler position) amino acid substitution alone does not confer resistance to ATM-AVI but is able to confer such resistance in the presence of truncated OmpK36 and frameshift mutation of *ramR*, which also could result in overexpression of the AcrAB-TolC efflux pump and decreased production of OmpK35 in *K. pneumonia*.^10^ Overexpression of the AcrAB-TolC efflux pump has also been found to confer resistance to ATM-AVI in combination with the production of DHA-1 (an AmpC cephalosporinase) and OmpK36 with mutation in *K. pneumonia*.^8^ However, whether overexpression of efflux pumps truly plays a role in mediating resistance to ATM-AVI remains to be determined owing to the co-existence of multiple other potential mechanisms in single strains. For CMY β-lactamases, there are eight AVI binding residues (S64, K67, E120, Y150, N152, K315, T316, and N346; one letter of amino acid and Ambler position). CMY with mutations occurring at these residues such as Y150C, Y150S and N346H could lead to ATM-AVI resistance alone in *E. coli* or in the presence of porin alteration in *K. pneumoniae* (Table 1).

In summary, ATM-AVI, which can be realized by combining ATM with CAZ-AVI at present, provides the full coverage for CRE producing MBLs and/or serine-β-lactamases. Currently-available limited clinical data suggest that ATM-AVI leads to favorable clinical outcomes and has been recommended as the first-line choice against MBL-producing CRE by well-recognized guidelines. Alarmingly, CRE resistant to ATM-AVI has also emerged. Resistance to ATM-AVI in Enterobacterales are multifactorial, mainly due to the combination of two or three of the following mechanisms: 1) enhanced hydrolysis to ATM and/or reduced binding to AVI due to overproduction or mutations of β-lactamases able to hydrolyze ATM; 2) decreased membrane permeability due to alterations, absence, or reduced production of porins such as OmpC/OmpF in *E. coli* or OmpK35/OmpK36 in *K. pneumoniae*; 3) target modification mainly due to modification of PBP3 in *E. coli*; and possibly 4) increased drug efflux. The clinical efficacy of ATM-AVI warrants further studies with large-scale patient sizes. Rigorous monitoring is also needed for ATM-AVI resistance in CRE.
